# OTX015 (MK-8628), a novel BET inhibitor, exhibits antitumor activity in non-small cell and small cell lung cancer models harboring different oncogenic mutations

**DOI:** 10.18632/oncotarget.13181

**Published:** 2016-11-07

**Authors:** Maria E. Riveiro, Lucile Astorgues-Xerri, Ramiro Vazquez, Roberta Frapolli, Ivo Kwee, Andrea Rinaldi, Elodie Odore, Keyvan Rezai, Mohamed Bekradda, Giorgio Inghirami, Maurizio D'Incalci, Kay Noel, Esteban Cvitkovic, Eric Raymond, Francesco Bertoni

**Affiliations:** ^1^ Oncology Therapeutic Development, Clichy, France; ^2^ IRCCS-Istituto di Ricerche Farmacologiche Mario Negri, Milan, Italy; ^3^ Lymphoma and Genomics Research Program, IOR Institute of Oncology Research, Bellinzona, Switzerland; ^4^ Dalle Molle Institute for Artificial Intelligence (IDSIA), Manno, Switzerland; ^5^ SIB Swiss Institute of Bioinformatics, Lausanne, Switzerland; ^6^ Radiopharmacology Department, Curie Institute - René Huguenin Hospital, Saint Cloud, France; ^7^ Department of Pathology and Laboratory Medicine, Weill Cornell Medical College, New York, NY, USA; ^8^ Department of Molecular Biotechnology and Health Science and Center for Experimental Research and Medical Studies (CeRMS), University of Turin, Turin, Italy; ^9^ Department of Pathology, and NYU Cancer Center, New York University School of Medicine, New York, NY, USA; ^10^ Oncoethix SA (now Oncoethix GmbH, a wholly owned subsidiary of Merck Sharp and Dohme Corp.), Lucerne, Switzerland; ^11^ Medical Oncology Department, CHUV, Lausanne, Switzerland; ^12^ Oncology Institute of Southern Switzerland (IOSI), Bellinzona, Switzerland

**Keywords:** OTX015 (MK-8628), bromodomain, NSCLC, SCLC, KRAS

## Abstract

Inhibitors targeting epigenetic control points of oncogenes offer a potential mean of blocking tumor progression in small cell and non-small cell lung carcinomas (SCLC, NSCLC). OTX015 (MK-8628) is a BET inhibitor selectively blocking BRD2/3/4. OTX015 was evaluated in a panel of NSCLC or SCLC models harboring different oncogenic mutations. Cell proliferation inhibition and cell cycle arrest were seen in sensitive NSCLC cells. *MYC* and *MYCN* were downregulated at both the mRNA and protein levels. In addition, OTX015-treatment significantly downregulated various stemness cell markers, including NANOG, Musashi-1, CD113 and EpCAM in H3122-tumors *in vivo*. Conversely, in SCLC models, weak antitumor activity was observed with OTX015, both *in vitro* and *in vivo*. No predictive biomarkers of OTX015 activity were identified in a large panel of candidate genes known to be affected by BET inhibition. In NSCLC models, OTX015 was equally active in both EML4-ALK positive and negative cell lines, whereas in SCLC models the presence of functional RB1 protein, which controls cell progression at G1, may be related to the final biological outcome of OTX015. Gene expression profiling in NSCLC and SCLC cell lines showed that OTX015 affects important genes and pathways with a very high overlapping between both sensitive and resistant cell lines. These data support the rationale for the OTX015 Phase Ib (NCT02259114) in solid tumors, where NSCLC patients with rearranged ALK gene or KRAS-positive mutations are currently being treated.

## INTRODUCTION

Lung cancer is the second most common cancer in adults and the main cause of cancer-related deaths [1]. Non-small cell lung carcinomas (NSCLC) account for approximately 80% of cases and include adenocarcinoma, squamous cell, and large cell carcinomas, while small cell lung carcinomas (SCLC) represent the other 20% of cases [2]. NSCLC and SCLC are distinct biologic, genetic and clinical diseases [2, 3]. Approximately 50%–60% of NSCLC patients have at least one identifiable driver mutation, with the most common mutations found in the Kirsten ras (*KRAS*) gene (25–30%) and the epidermal growth factor receptor (*EGFR*) gene (13–22%), along with translocations involving anaplastic lymphoma kinase (*ALK*) gene in another 5–6% cases [2–5]. On the other hand, SCLC is mainly characterized by the prevalence of inactivating mutations in tumor suppressor genes such as *TP53*, *PTEN*, and *RB1*, and activation of MYC family members [3].

The bromodomain (BRD) and extraterminal (BET) family are protein interaction modules that specifically recognize Ɛ-N-acetylated lysine residues [6, 7], composed of three ubiquitously expressed proteins, BRD2, BRD3 and BRD4, as well as the testis-specific BRDT. BRDs are common in nuclear proteins that regulate gene transcription and chromatin organization, and play a key function in recruiting these protein complexes to acetylated chromatin. Dysfunction of BRD-containing proteins has been linked to the development of cancer [8, 9] and research has recently focused on the therapeutic efficacy of a novel class of epigenetic compounds that selectively target BET proteins in human malignancies [10–12]. OTX015 (MK-8628) is a selective thienotriazolodiazepine BET inhibitor that competitively occupies the acetyl-binding pockets of BRD2/3/4, resulting in their release from active chromatin and suppression of downstream signaling to RNA polymerases [13–15]. OTX015 has shown potent *in vitro* and *in vivo* antitumor activity against a range of cell lines derived from hematologic malignancies [14, 16] and solid tumors including neuroblastoma, glioblastoma, breast and prostate cancers [15, 17–20]. In some models, OTX015 biological activity was shown to modulate transcriptional pathways characteristic of MYC functioning, however overt effects on other key transcriptional pathways such as AP-1 and NF-kB were also observed [14]. An inhibitory effect on *MYCN* function has been reported in translational models of neuroblastoma [15]. OTX015 is the first BET inhibitor to have moved into the clinic, with three phase Ib clinical trials initiated in hematologic malignancies (NCT01713582) [21, 22], selected solid tumors (NCT02259114) and glioblastoma multiforme (NCT02296476).

We report here preclinical findings of the BET inhibitor OTX015 in NSCLC and SCLC cell lines harboring oncogenic mutations recurrently found in lung cancer patients. In NSCLC models, OTX015 was equally active in both EML4-ALK positive and negative cell lines harboring other oncogenic mutations. OTX015-exposure resulted in rapid and sustained downregulation of MYC or MYCN, together with an *in vivo* downregulation of stemness markers in sensitive NSCLC models. Conversely, we observed that despite broad amplification of MYC family genes, OTX015 did not show potent *in vitro* or *in vivo* antitumor effects in the SCLC models evaluated.

## RESULTS

### OTX015 reduces cell proliferation and induces cell cycle arrest in NSCLC cell lines with or without the EML4-ALK translocation

OTX015 displayed antiproliferative effects in EML4-ALK negative and positive NSCLC cell lines (Table 1, detailed in Supplementary Table S2). After 72 h exposure, two out five cell lines (HOP62, HOP92) displayed GI50 values below 0.5 μM, whereas H2228 and H3122 cells presented GI_50_ values below 1.0 μM (0.63 and 0.70 μM, respectively). In addition, these four cell lines displayed E_max_ values from 35% to 58% after 72 h-exposure. On the other hand, A549 cells presented a GI_50_ > 6 μM and an E_max_ of 82%. The OTX015-resistant A549 cell line presents both KRAS and LKB1 mutated genes (Table 1). OTX015 was more potent than JQ1 following 72 h-exposure in all five cell lines. OTX015 inhibited cell proliferation in sensitive cell lines at *in vitro* concentrations that are achievable in plasma samples of patients treated with nontoxic OTX015 doses in an ongoing Phase I study in hematologic malignancies [23].

**Table 1:** Antiproliferative effects of the BET inhibitor OTX015 in NSCLC and SCLC cell lines

Tumor type	Cell line	OTX015		JQ1										
		GI50 [μM] (95% IC)	Emax (at 6 μM)	GI50 [μM] (95% IC)	Emax (at 6 μM)	KRAS Exon2	EGFR Exon 20	PIK3CA Exon 20	LKB1	TP53	RB1	EML4-ALK fusion protein	MYC Amplification	MYCN Amplification
NSCLC	**HOP92**	0.11 (0.08–0.17)	54	0.38 (0.31–0.47)	55							—	Y	–
NSCLC	**HOP62**	0.10 (0.06–0.16)	58	0.42 (0.38–0.59)	59							—	—	—
NSCLC	**A549**	> 6	82	> 6	74							—	—	—
NSCLC	**NCI-H2228**	0.63 (0.42–0.95)	35	3.42 (1.31–8.92)	w							Y (Var 3)	NEii	NE
NSCLC	**NCI-H3122**	0.70 (0.52–0.93)	41	2.87 (1.89–4.37)	22							Y (Var 1)	NE	NE
SCLC	**NCI-H69**	> 6	87	1.03 (0.51–2.07)	53							—	—	Y
SCLC	**NCI-H82**	> 6	92	> 6	89							—	Y	—
SCLC	**DMS-79**	> 6	84	> 6	83							—	Y	ii
SCLC	**DMS-114**	0.12 (0.08–0.17)	54	0.79 (0.51–1.24)	51							—	—	—

GI50 and Emax (at 6 μM) values for OTX015 and JQ1 after 72h-exposure, by MTT assay. Results represent the mean and 95%CI of at least three independent experiments performed in triplicate. The cell panel was characterized for *KRAS*, *EGFR*, *PIK3CA*, *LKB1*, *TP53*, *RB1*, *EML4-ALK* fusion protein and *MYC* and *MYCN* amplification. Red indicates mutation, blue is wild-type, Y = yes, and NE = not evaluated.

**Figure 1:**
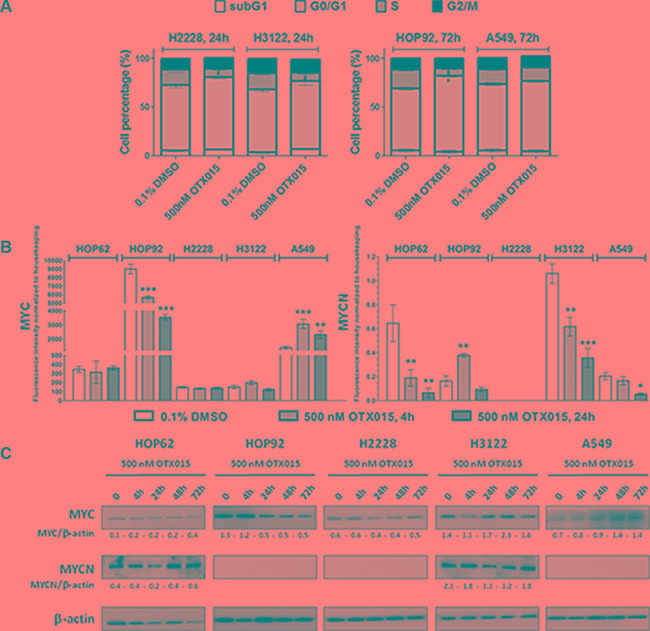
**(A)** OTX015 induces cell cycle changes in OTX015-sensitive NSCLC cell lines. Effect of 500 nM OTX015 on cell cycle progression after 24 h in H2228 and H3122 and after 72 h in HOP92 and A549 cells, by FACScan, expressed as percent cells per cell cycle phase (*p < 0.05 for G0/G1 cell cycle phase, and ^#^*p* < 0.05 for S phase). (**B**) OTX015 modulates MYC and MYCN mRNA levels in sensitive and resistant NSCLC cell lines. Effect of 500 nM OTX015 on MYC and MYCN mRNA levels after 4 and 24 h by qPCR, expressed as fluorescence intensity normalized to housekeeping genes. Results represent the mean ± SD of one representative experiment performed in duplicate (*p < 0.05, **p < 0.01, ***p < 0.001 respect to controls). (**C**) OTX015 effects on MYC and MYCN protein levels in NSCLC cell lines by Western blot. Cells were exposed to 500 nM OTX015 for up to 72 h. Results are representative of at least two independent experiments. β-actin was used as a loading control and densitometry analysis was done using Image J software.

To determine if OTX015 exerts cytostatic effects in NSCLC cells, as previously described for other adult cancers [14, 17, 20] we evaluated cell cycle regulation after 500 nM OTX015 treatment in three OTX015-sensitive cell lines (HOP92, H2228 and H3122) and one resistant model (A549). After 24 h-treatment a decrease in cells in the S phase was seen in H2228 and H3122 cell lines, while then after 72 h of OTX015-exposure in HOP92 cells a significant increase in the percentage of cells in G1, along with a decrease in the percentage of cells in the S phase were seen (*p* < 0.05) (Figure 1A) after 72 h-treatment. No modulation of cell cycle phases was observed at any time point for the OTX015-resistant cell line A549. Similar results were obtained with JQ1 (data not shown).

The effect of 500 nM OTX015 on mRNA levels of MYC and MYCN were evaluated in the four OTX015-sensitive and one resistant cell lines. As shown in Figure 1B, OTX015 treatment resulted in a rapid and sustained downregulation of MYC in HOP92 cells. MYCN was downregulated in HOP62 and H3122 after 4 and 24 h, while in the resistant cell line, A549 after 24 h only. On the other hand, it was upregulated in HOP92 after 4 h but downregulated at 24 h (Figure 1B). At the protein level, MYC was downregulated after 24 h in the MYC amplified cell line HOP92, which was maintained up to 72 h (Figure 1C). MYCN protein was only detected in HOP62 and H3122 cell lines, where a transient decrease was observed after 24 h of treatment with recovery to baseline levels by 72 h in H3122 cells, whereas an increase was seen in HOP62 cells after 72 h.

The four OTX015-sensitive cell lines and one OTX015-resistant cell line were analyzed for putative predictive biomarkers of OTX015 activity. We first evaluated potential correlations between basal mRNA and protein levels of a panel of candidate markers. Basal mRNA levels of 14 genes implicated in BET signaling pathways and whose expression is known to be affected by BET inhibition [14, 31, 32] were evaluated: BRD2/3/4, MYC and MYCN, CDKN1A (P21), BCL2, HEXIM1, SESN3, MTHFD1L, and four genes coding for histones (HIST2H2BE, HIST1H2BJ, HIST1H2BK and HIST2H4A). At baseline, the five cell lines expressed detectable and widely variable levels of these 14 genes, without a clear correlation with OTX015 sensitivity (Supplementary Figure S1A). Analysis of basal protein levels in the five NSCLC cell lines also failed to reveal any clear correlations between OTX015 sensitivity and basal protein levels of BRD2, BRD3, BRD4, MYC, MYCN, cyclin D1, P21, BCL2 or p-^Tyr705^STAT3 (Supplementary Figure S1B).

A correlation analysis between gene expression changes of the 14 genes after OTX015 treatment with drug sensitivity (E_max_) did not show any identify any predictive markers in the NSCLC panel. Modulation of MYC, BRDs, HEXIM1, BCL2, CDKN1A, MTHFD1L, SESN3, HIST1H2BJ, HIST2H2BE and HIST1H2BK mRNA levels after 24 h with 500 nM OTX015 showed no correlative tendencies with OTX015 biological outcomes in our panel of sensitive and resistant NSCLC cell lines (Supplementary Figure S2A). Likewise, no correlations were apparent after 4 h for any of the genes evaluated (data not shown).

### OTX015 abrogates *in vivo* growth of EML4-ALK (+) NSCLC tumors and downregulates MYC genes and stem cell markers

**Figure 2:**
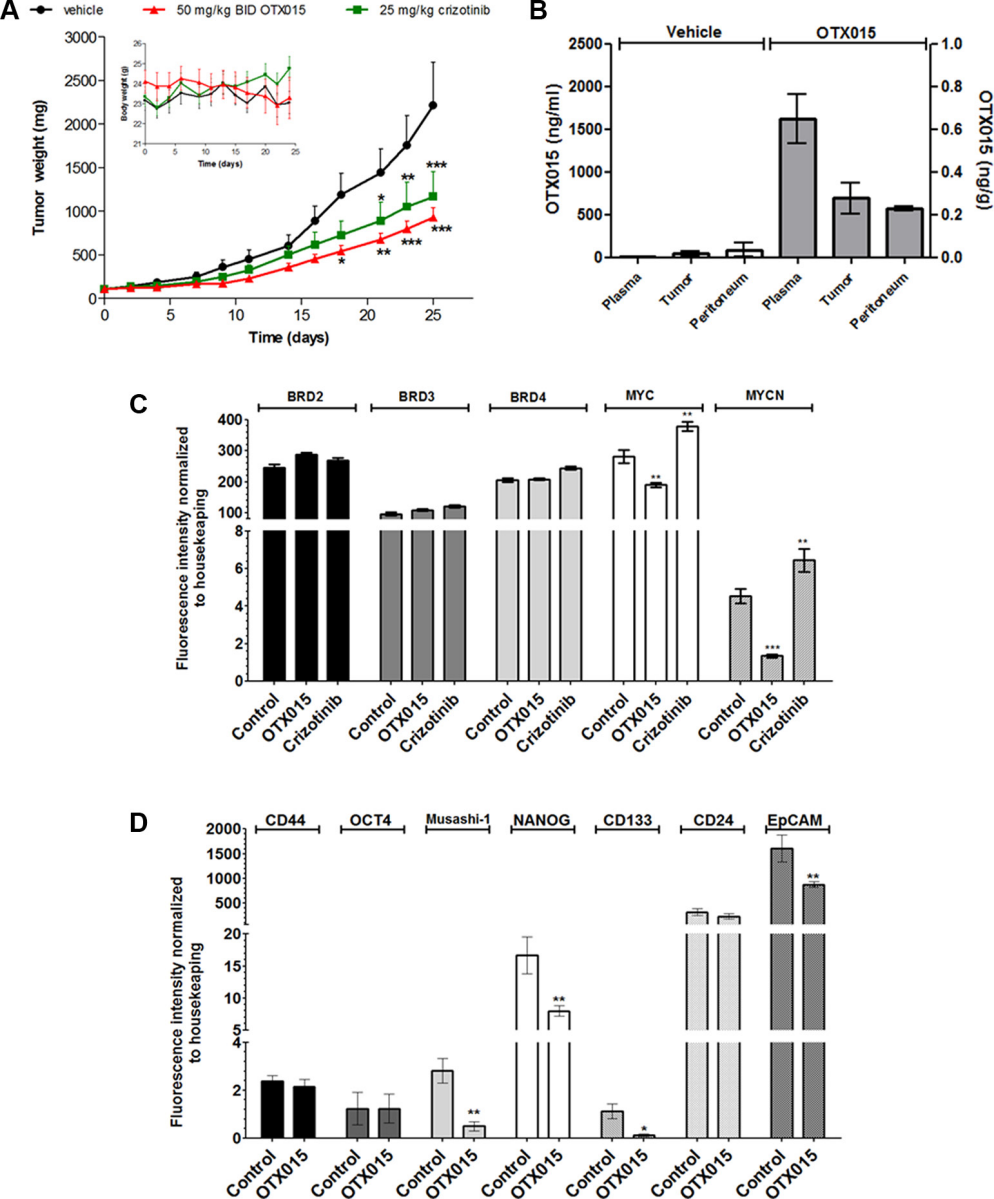
(A) OTX015 *in vivo* effects in NSLCL-ALK(+) H3122-tumor bearing mice. Antitumor effects of 50 mg/kg OTX015, BID, 7 days ON, gavage or 25 mg/kg crizotinib, thrice weekly, gavage were compared with vehicle thrice weekly, gavage in H3122 murine xenografts (*n* = 10 mice per group). Asterisks indicate significant differences in tumor mass between each single agent arm versus the vehicle-treated group (*p < 0.05, **p <0.01 and ***p < 0.001). (**B**) OTX015 concentrations in plasma and tissue from H3122 xenografts. OTX015 levels were evaluated in terminal plasma, peritumoral normal and tumor tissue from H3122-bearing mice treated with 50 mg/kg OTX015 BID or vehicle for 25 days. Mice were sacrificed 4 h after the last administration. Results are expressed as mean ± SD. (**C)** Gene expression of BRDs and MYC family genes by qPCR in H3122 tumors after treatment with OTX015. Differences in gene expression between OTX015 (50 mg/kg OTX015) and crizotinib (25 mg/kg thrice weekly) with respect to controls were evaluated with one-way Anova followed by Dunnett's Multiple Comparison Test (**p < 0.01 and ***p < 0.001). (**D**) Gene expression of stem cell markers by qPCR in H3122 tumors after treatment with OTX015. Differences in gene expression between OTX015 and crizotinib with respect to controls were evaluated with one-way Anova followed by Dunnett's Multiple Comparison Test (*p < 0.05 and **p < 0.01).

In H3122 xenografts, a significant reduction in tumor growth was observed with 50 mg/kg OTX015 BID and crizotinib 25 mg/kg thrice weekly (*p* < 0.001 at day 25 with respect to vehicle for both agents) (Figure 2A). On the basis of T/C%, OTX015 but not crizotinib was considered active (maximum T/C 42% and 53% on day 24, respectively). No significant weight loss or overt signs of toxicity were observed with OTX015 or crizotinib (Figure 2A, insert), with both treatments generally well tolerated. OTX015 levels were determined in plasma, tumor and normal muscle tissues of control and treated mice. Plasma concentrations were over 500 nM, while slightly lower levels were found in tumor and normal tissue (~300 nM; Figure 2B).

Analysis of tumor gene expression in H3122-bearing mice at the end of treatment did not show significant variations for any of the OTX015 canonical targets BRD2, 3 and 4 with respect to vehicle-treated animals (Figure 2C), as observed after *in vitro* analyses in NSCLC cell lines following OTX015 exposure (data not shown). On the other hand, OTX015 treatment significantly decreased MYC and MYCN mRNA levels in H3122-tumors (*p* < 0.01 and *p* < 0.001). Conversely, we observed an increase in levels of these MYC family proteins in crizotinib-treated tumors. In light of these findings, and since most NSCLC-ALK-positive patients acquire resistance to crizotinib within a few months [5], we explored the combination of OTX015 with the ALK inhibitor crizotinib. Concomitant combination of OTX015 with crizotinib showed additive effects (CI ≤ 1.1) after 48 h in two EML4-ALK-positive NSCLC cell lines (H2228 and H3122) (Supplementary Figure S3).

We also evaluated the effect of OTX015 on a panel of stemness markers *in vivo*, observing that OTX015 induced significant transcriptional downregulation of NANOG, as well as Musashi-1, CD113 and EpCAM in NSCLC tumors, while it did not have an effect on other pluripotency genes such as Oct4, CD44 and CD24 (Figure 2D).

### OTX015 shows limited *in vitro* and *in vivo* activity in SCLC models, with MYC modulation in sensitive and resistant cell lines

**Figure 3:**
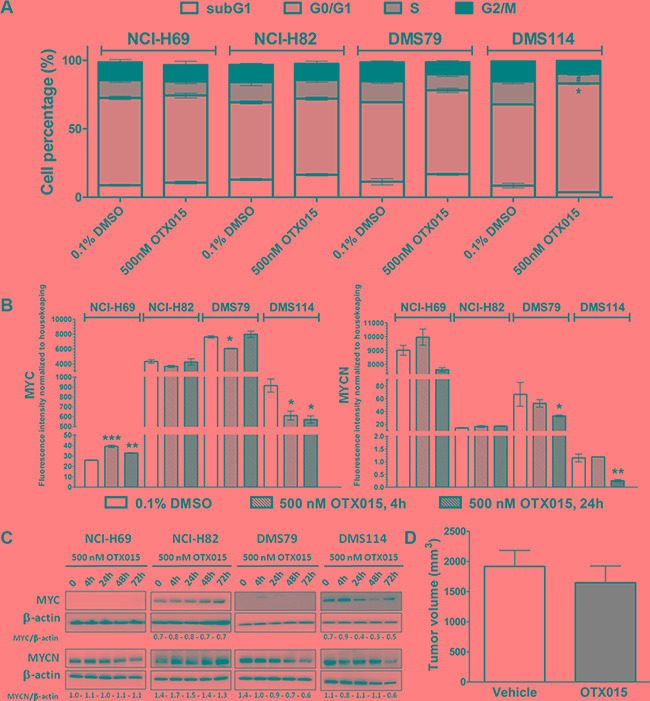
(A) OTX015 induces cell cycle changes in OTX015-sensitive SCLC DMS114 cells. Effect of 500 nM OTX015 on cell cycle progression after 72 h, by FACScan, expressed as percent cells per cell cycle phase (*p < 0.05 for G0/G1 cell cycle phase, and ^#^*p* < 0.05 for S phase). (**B**) OTX015 modulates MYC and MYCN mRNA levels in sensitive and resistant SCLC cell lines. Effect of 500 nM OTX015 on MYC and MYCN mRNA levels after 4 and 24 h, by qPCR, expressed as fluorescence intensity normalized to housekeeping genes. Results represent the mean ± SD of one representative experiment performed in duplicate (*p < 0.05, **p < 0.01, ***p < 0.001 respect to controls). (**C**) OTX015 effects on MYC and MYCN protein levels in SCLC cell lines by Western blot. Cells were exposed to 500 nM OTX015 for up to 72 h. Results are representative of at least two independent experiments. β-actin was used as a loading control and densitometry analysis was done using Image J software. (**D**) OTX015 *in vivo* effects on tumor volume in DMS114 murine xenografts. Tumor volume was determined after 28 days of treatment with vehicle or 50 mg/kg OTX015, BID, 7 days ON, gavage (*n* = 10 mice per group).

Among the four SCLC models evaluated, only the DMS114 cell line had an OTX015 GI_50_ value below 0.5 μM after 72 h-exposure, with all other cell lines being resistant (Table 1). Interestingly, we observed that SCLC OTX015 resistant cells displayed mutation for RB1 gene, mutation which was not observed in the sensitive one DMS114 (Table 1). Similar results were seen with OTX015 and JQ1 except for NCI-H69 which was more sensitive to JQ1 (Table 1). To determine if OTX015 exerts cytostatic effects in DMS114 cells, we evaluated cell cycle progression after 72 h OTX015 (500 nM) (Figure 3A), which revealed a significant increase in the percentage of DMS114 cells in the G0/G1 phase, along with a significant decrease in the number of cells in the S phase, with no effect detected in the other SCLC cell lines.

Analysis of potential downstream targets showed that OTX015 resulted in rapid and sustained downregulation of MYC in the OTX015-sensitive cell line DMS114 (Figure 3B), whereas a sustained upregulation of MYC was observed in NCI-H69 cells. On other hand, no significant modulations were seen in MYCN after 4 h, while it was downregulated in NCI-H69, DMS79 and DMS114 cells after 24 h (Figure 3B). At the protein level, MYC expression was downregulated after 24 h in DMS114 cells, which was maintained through to 72 h. In NCI-H69, DSM79 and DMS114 cells, MYCN protein levels were downregulated after 48 h-exposure (Figure 3C).

As observed in NSCLC cell lines, no correlations were identified between the basal mRNA expression of BRD2, 3 or 4, histones (HIST2H2BE, HIST1H2BJ, HIST1H2BK and HIST2H4A), MYC, MYCN, BCL2, P21, HEXIM1, SESN3 and MTHFD1L nor after 24 h with OTX015 in SCLC cell lines (Supplementary Figure S2B). This should be considered in light of the fact that only one of the four cell lines evaluated was sensitive to OTX015 *in vitro*.

Although OTX015 presented good oral bioavailability and pharmacokinetic properties in the *in vivo* murine xenografts (data not shown), no significant differences in tumor volume were seen between OTX015 and vehicle mice after 28 days of *in vivo* OTX015 treatment (Figure 3D). The median time for tumors to reach 1000 mm^3^ was 14.1 and 19.3 days for the vehicle and OTX015-treated arms, respectively.

### OTX015 affects key biologic pathways in sensitive NSCLC and SCLC cells

**Figure 4:**
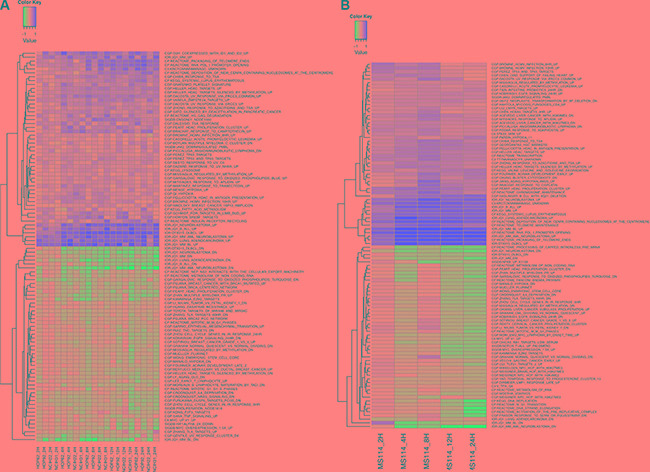
Heatmap of the average gene set expression of the most enriched gene sets among genes changing after exposure to OTX015 in lung cancer OTX015 sensitive cell lines (**A**) NSCLC cell lines; (**B**) SCLC cell line. The top 50 most enriched gene sets among upregulated genes and the top 50 enriched gene sets among downregulated genes are shown. Gene set expression was calculated using GSVA. Y-axis: cell lines at different time points. The heatmap colors are not scaled and show the consistent increase (or decrease) of gene set expression in time. X-axis: top differentially expressed gene sets as assessed by GSEA. Gene sets were filtered for a minimal fold-change of > 0.15 and FDR < 0.05.

Gene expression changes were evaluated by GEP in sensitive and resistant lung cancer cell lines exposed to OTX015 (500 nM) or DMSO for 2, 4, 8, 12 or 24 h. OTX015 affected key biologic pathways in a time-dependent manner in both NSCLC sensitive and resistant cell lines (Figure 4, Supplementary Figures S4–S8, Supplementary Tables S3–S4). Downregulated transcripts were enriched for genes involved in cell cycle and apoptosis regulation, the NFKB/IFN/MYD88 pathway, as well as MYC, STAT and MIR34b/C targets. Upregulated transcripts were enriched for genes involved in chromatin structure and TP53/TP63 targets. The most differentially expressed genes after OTX015 exposure included different class 1 and 2 histones-coding genes, *IRF7*, *EFR3B*, *FOS*, *EGR1*, *FGFR3*, *HES6*, *NOTCH1*, *ID1*, *KLF2*, *GADD451*, *CDKN1A*, *BCL6*, *SERTAD1, XEXIM1, SESN3* and *CDKN1A* among the up-regulated and *CCL2*, *IL7R*, *IL8*, *IL6*, *TP63*, *MYC*, *BCL2L1*, *BIRC3*, *JUND*, *TGFB2*, *BAMBI*, *AURKA*, *TFPI*, *HMG1A*, *JUND*, *MTHFD1L, MLKL*, *DKK1*, *VEGFC, PDGFC* and *E2F2* among the down-regulated (Supplementary Tables S3–S4). Unexpectedly based on the differences in term of GI50 values, these genes were largely overlapping with those changing also in the resistant NSCLC cells, but individual transcripts behaving differently could be identified. Resistant cells presented a reduced modulation of *CDKN1A* (less up-regulated), *IL7R*, *IL6*, *CSF2*, *RUNX2* (less down-regulated) [14, 26, 27], and, in accordance with the above-mentioned protein data, the early down-regulation of *MYC* was not observed in the resistant setting but only in the sensitive cell lines. Genes coding for proteins supporting cancer cells survival or proliferation such as *FOXD1* or *EIF5A* appeared up-regulated in resistant and not in sensitive, while E-cadherin, *CLDN7*, *MDK*, *PKDCC*, and *JAG1* were more down-regulated.

In SCLC cell lines, OTX015 also affected relevant biologic pathways and individual transcripts in a time-dependent manner (Figure 4, Supplementary Figures S4–S6 and S9–S10, Supplementary Tables S4–S5). Downregulated transcripts were enriched for genes involved in cell cycle and apoptosis regulation, NFKB/MYD88 pathways, as well as MYC and STAT4/5 targets. Upregulated transcripts were enriched for genes involved in chromatin structure and TP53/TP63 targets. The most up-regulated genes after exposure to OTX015 comprised genes coding for different class 1 and 2 histones, *IRF7*, *CDKN1A*, *CDKN1C*, *BRD2*, *XEXIM1*, *SESN3*, *STAT3* *FGFR3*, *MKNK2* and *EGR1*, while the most down-regulated transcripts encompassed *E2F2*, *EIF5A*, *DKK1*, *CCL2*, *TPM3*, *CA2*, *MUC1*, *KRAS*, *VEGFB*, *MTHFD1L*, *BCL2L1*, *AURKA*, *PDGFC*, and *PDE4D* (Supplementary Tables S4–S5). Again, GEP signatures largely overlapped between sensitive and resistant cells (Supplementary Figures S9–S10). Since only one sensitive and one resistant SCLC cell lines were studied, the vast majority of differences might be due to the cell of origin. However, we could observe a different regulation of *CDKN2D* (P19), up-regulated in resistant cells, and of *CDKN1C* (P57), up-regulated in the sensitive cells.

Finally, OTX015-induced changes in NSCLC and SCLC models strongly overlapped with OTX015 signatures in diffuse large B cell lymphomas [14], with JQ1 signatures in NSCLC [24] and other tumor models [26, 28–30], and also partially overlapped with those of HDAC inhibitors (Figure 4, Supplementary Figures S4–S6).

## DISCUSSION

In this study, a panel of lung cancer models harboring a range of oncogenic mutations, MYC amplifications, and the fusion protein EML4-ALK, representing the most relevant clinical subtypes of NSCLC or SCLC were selected to evaluate the biological relevance of BET inhibition in lung cancer and to better define which subtypes have the potential to be more sensitive to BET inhibition. We hereby provide supporting evidence to explore this new therapeutic avenue of BET proteins inhibition in lung cancer.

OTX015 demonstrated a range of antitumor activity with GI_50_ values in the submicromolar range in several NSCLC and SCLC models, displaying cytostatic activity. OTX015 treatment resulted in an accumulation of cells in the G1 phase and depletion of cells in the S phase in OTX015-sensitive cell lines. Our data were similar to what was reported with the OTX015 analog JQ1 in lung cancer models [24, 31, 32], although, in our hands, OTX015 presented lower GI_50_ values than JQ1.

Initially, cells harboring the EML4-ALK fusion protein were expected to be more sensitive to BET inhibition, which is known to be implicated in the dependency between the ALK and MYC pathways [33]. However, four NSCLC cell lines were found to be equally susceptible to BET inhibition independent of their *KRAS* and *EGFR* mutational status and of the chimeric oncogene EML4-ALK. These results were in accordance with previous studies showing that the presence of *EGFR* mutations does not affect BET activity [32]. In addition, our findings with the A549 cell line were coherent with a previous report showing that KRAS/LKB1 mutant models are resistant to JQ1 [32]. In sensitive NSCLC models, OTX015 resulted in a rapid and sustained downregulation of MYC (HOP92) or a transient MYCN downregulation (HOP62 and H3122). Given that MYC or MYCN were not altered by OTX015 treatment in the OTX015-sensitive H2228 cell line, it will be valuable to investigate other proto-oncogenes such as MYCL1, or FOSL1 for a potential role in the OTX015 mechanism of action, as described previously for JQ1 [24]. *In vivo* experiments confirmed that OTX015 prompted tumor regression in an EML4-ALK(+) NSCLC model, modulating both MYC and MYCN together with the down-regulation of several stem cells markers, in particular NANOG as well as Musashi-1, CD113 and EpCAM in NSCLC tumors, whereas little effect on other pluripotency genes such as Oct4, CD44 and CD24 was observed. In support of these findings, it was recently shown that BRD4 is critical for maintenance of stem cell pluripotency which primarily occurs through the regulation of NANOG expression with only minimal effects on other pluripotency genes such as Sox2, Oct4, and Klf4, and JQ1 exposure inhibits the BRD4 binding to NANOG promoter leading to a marked transcriptional downregulation of the expression of the gene, without affecting MYC [11, 34].

Despite initial response to crizotinib, the majority of patients with ALK-rearranged tumors, relapse within 12 months and develop resistance [35]. Treatment options after failure with crizotinib are limited. Here, OTX015 was more potent than crizotinib in the *in vivo* setting without apparent toxicity and additive effects were seen when the two agents were combined *in vitro* in two EML4-ALK(+) NSCLC models. Interestingly, we observed that *in vivo,* in contrast to OTX015, crizotinib upregulated MYC and MYCN transcripts. Studies exploring the positioning of OTX015 as second-line treatment after crizotinib-acquired resistance or for delaying crizotinib resistance appearance are merited.

Based on the relevance of deregulation of MYC family members in SCLC [36], we hypothesized that SCLC models would be more susceptible to BRD inhibition/MYC gene downregulation than other tumor types. Instead, we observed that OTX015 treatment only decreased MYC mRNA and protein levels in DMS114 cells, whereas the three other SCLC models were resistant to OTX015 (GI_50_ > 6 μM after 72 h) suggesting that sensitivity to OTX015 is not dependent on MYC modulation. In addition, no correlations were observed between SCLC cell lines harboring MYC gene amplification and OTX015 sensitivity. DMS114 cells harbor a wildtype *RB1* gene while the three OTX015-resistant cell lines carry an inactive *RB1* mutant, which may be implicated in the outcome of BET inhibition in this type of lung tumor. This would be in accordance with the observation that lymphoma cell lines with a strong E2F gene expression signature present lower sensitivity to OTX015 [14].

Transcriptome analysis after OTX015 treatment revealed wide-ranging modulation in genes coding for several histones and several genes involved in cell cycle, cell growth, apoptosis and migration, surprisingly in both OTX015-sensitive and OTX015-resistant cell lines, and for both NSCLC and SCLC models. OTX015 signatures also largely overlapped with what reported for the same compound or for other BET inhibitors in different tumor models.

The observed gene expression changes were very similar to what observed with OTX015 or with other BET inhibitors in other tumor models [14, 15, 26, 30, 37]. OTX015 down-regulated genes involved in cell cycle and apoptosis regulation, NFKB/MYD88 pathways, as well as MYC targets, while the genes coding for proteins involved in chromatin structure were the most commonly up-regulated genes. The down-regulation induced by OTX015 on a series of individual transcripts might have positive clinical implications for patients with lung cancer: *AURKA* [38], *KRAS*, *MYC* [36], *CDK6* [39], *MUC1* [40], *IL6* [41], *CCL2* [42], *IL7R* [43], *E2F2* [44], *TGFB2* (its receptor *BAMBI* was up-regulated after OTX015) [45], *PDE4D* [46], *TPM3* [47], and *CA2* [48].

Analysis of the gene expression changes after OTX015 also lead to two unexpected observations. First, exposure to OTX015 led to the up-regulation of genes that might reduce the anti-tumor activity of the BET inhibitor such as *STAT3* and *FGFR3* in both NSCLC and SCLC, *NOTCH1* and its target *HES6* in NSCLC, *PIM2* in SCLCL: further studies are needed to understand the relationship between these genes and OTX015 and whether novel combinations with agents targeting these proteins can improve the response to the epigenetic compound. Second, despite big differences in terms of GI_50_ values, sensitive and resistant cell lines presented very similar gene expression changes after exposure to OTX015 given at 500 nM. Although our analyses encompassed only a limited number of resistant lung cancer cell lines, a few hints could be made on the mechanisms underlying primary resistance to OTX015. NSCLC resistant cells presented a reduced modulation of known BET inhibitors targets [14, 26, 27] such CDKN1A (less up-regulated), IL7R, IL6, CSF2, RUNX2 (less down-regulated), and, in accordance with our protein data, the early down-regulation of MYC was observed only in the sensitive cell lines. After OTX015 SCLC resistant and sensitive cells differed presenting an up-regulation of CDKN2D (P19) in resistant cells, and of CDKN1C (P57) in the sensitive cells, changes that could be linked with the different sensitivity to anti-cancer drugs [49, 50].

Our findings indicate that the subsets of NSCLC cells harboring the EML4-ALK chimeric oncogene appear sensitive to BET inhibition. Also, concurrent mutations in *KRAS* and *LKB1* (coding a serine-threonine kinase that directly phosphorylates and activates AMPK) genes abrogated OTX015 effects in NSCLC, as previously described for JQ1 [32]. In SCLC models, our findings highlighted that absence of expression of the product of *RB1* (the retinoblastoma protein, pRB) as a consequence of deletion, mutation, chromosomal loss or other mechanisms, as is the case in almost 90% of SCLC patients, might be a resistance marker for BET inhibition in SCLC. In contrast to direct small-molecule inhibitors of driver oncoproteins where specific DNA mutations serve as reliable biomarkers, the establishment of predictive markers of BET inhibition is heavily affected by the underlying heterogeneous genetic and epigenetic landscape of human cancers, including lung cancers, that can lead to the activation of different driver genes in specific individual cancers, or even in different subclones of the same tumor [51]. BET bromodomain proteins can be expected to have different targets in cancers derived from different cells, and this may influence the activity and mechanism of action of BET inhibitors. Collectively, our findings indicate that the cellular context-dependent requirement of BET inhibition cannot be fully explained on the basis of the known molecular functions of BRDs, as also highlighted by largely overlapping gene expression changes in both sensitive and resistant cell lines, or the basal levels of MYC genes, and to date, no genetic, transcriptional or chromatin markers have been identified to predict responsiveness to BET inhibition, except the concurrent mutations in *KRAS* and *LKB1* genes in lung carcinoma models [32].

In conclusion, we reported a broad-spectrum activity of OTX015 against NSCLC cell lines, but not in SCLC. OTX015 appears to be a promising therapeutic strategy for EML4-ALK positive patients as well as *KRAS*-mutant NSCLC patients with wildtype *LKB1*, whereas *EGFR* mutations did not correlate with the outcome of BET inhibition. In addition, OTX015 presented good oral bioavailability and induced tumor regression in EML4-ALK(+) xenografts. It inhibited MYC expression however this was not related to its sensitivity in cancer cell lines. In addition, we provide a rationale for investigating OTX015 as a single agent and combined with crizotinib in NSCLC-ALK(+) patients. These findings were the scientific support for the OTX015 dose escalation study in solid tumors including NSCLC patients (NCT02259114).

## MATERIALS AND METHODS

### Cell culture and reagents

OTX015 (MK-8628) was provided as a powder by Oncoethix. It was dissolved in DMSO as a 10 mM stock solution and stored at −20°C. JQ1 was purchased from BPS Bioscience. Crizotinib, a protein tyrosine kinase inhibitor of the MET receptor and ALK-fusion protein, was purchased from Selleck Chem. Drug stock solutions were reconstituted according to supplier instructions. Reagents were purchased from Sigma-Aldrich unless otherwise specified.

H2228, NCI-H69, NCI-H82 and DMS79 cell lines were obtained from the American Type Culture Collection, HOP92, HOP62, A549 and DMS114 from the National Institute of Cancer, Bethesda, USA, while the H3122 cell line was a kind gift of Prof. Giorgio Inghirami. Cell lines were maintained in T-25 culture flasks in RPMI 1640 supplemented with 10% FBS (#26140079, Gibco FBS qualified USA origin, Life Technologies), 2 mM glutamine (PAA Laboratories), 100 units/ml penicillin and 100 μg/ml streptomycin (PAA Laboratories) at 37°C in a humidified 5% CO2 atmosphere. Cell lines with a passage number ranging from 5 and 40 were used and were maintained in culture for up to 6 weeks. Confirmation of cell identity and verification of *Mycoplasma sp.* contamination was performed by Idexx BioResearch (Germany). Cell counting was performed with a hemocytometer.

### Cell cycle analyses

5 × 10^5^ cells were treated with 500 nM OTX015, JQ1 or equivalent concentrations of DMSO for 48 h, then stained with citrate buffer RNase (1 mg/ml) and propidium iodide (100 μg/ml) for 15 minutes at room temperature. DNA content and cell cycle distribution were analyzed by FACScan. Experiments were independently performed at least three times, unless otherwise indicated.

### *In vivo* studies

6-week-old female nude Foxn1 mice were obtained from Harlan Laboratories (Udine, Italy) and maintained at a constant temperature and humidity, according to institutional guidelines. Protocols were approved by the ethics committee of the IRCCS-Istituto di Ricerche Farmacologiche Mario Negri, Italy. For the NSCLC model, mice were subcutaneously injected in the right flank with the H3122 EML4-ALK-positive cell line (10^7^ cells/mouse). When tumors reached ~100 mg, mice were randomized to 1) vehicle: 0.5% methocel, thrice weekly, gavage; 2) 50 mg/kg OTX015, bi-daily (BID), 7 days ON, gavage; or 3) 25 mg/kg crizotinib, thrice weekly, gavage. For the SCLC model, DMS114 tumor fragments were subcutaneously grafted in the right flank. When tumors reached ~100–150 mg, mice were randomized to: 1) vehicle (PBS, gavage) or 2) 50 mg/kg OTX015, BID, 7 days ON, gavage. Tumor size measurements and mouse body weight were recorded at least twice a week from treatment start until the end of the study. Treatment efficacy was calculated as T/C%, where T and C are the mean tumor weight of treated and control groups, respectively. A T/C% < 45% was considered active. Tolerability was evaluated on the basis of body weight loss, clinical observation and mortality.

### Quantification of OTX015 levels in plasma and tissue samples

OTX015 concentrations were determined using a validated Acquity Ultra Performance Liquid Chromatography System (Waters) coupled with a tandem mass spectrometry detection method (UPLC/MS/MS), as described previously [52]. *In vivo* OTX015 levels were analyzed in terminal plasma, peritumoral tissue, peripheral and tumor tissues from H3122 xenograft-bearing nude mice. Control and OTX015-treated mice were sacrificed 4 h after the last treatment.

### Gene expression profiling (GEP)

RNA samples isolated using Trizol extraction from 2 × 10^6^ cells treated with 500 nM OTX015 or 0.1% DMSO were processed using the Human HT-12 v4 Expression BeadChip (Illumina), as previously described [14]. Data processing and statistical analysis were performed using R/Bioconductor. Transcript mapping was based on HG19 using manufacturer supplied annotation. Data were quantile normalized and differential expression analysis was performed using limma [53]. Functional annotation was performed using gene set enrichment analysis (GSEA) method using the GSEA gene sets [54], the Signature Database [55], and previously reported gene sets [14]. Differential expression of gene sets was calculated using the gene set variation analysis (GSVA) method [56]. The GEP data have been deposited in NCBI's Gene Expression Omnibus [57] and are accessible through GEO Series accession number GSE72961 (https://www.ncbi.nlm.nih.gov/geo/query/acc.cgi?acc = GSE72961).

### Chou-Talalay combination studies

For combination studies, 20 × 10^3^ cells/well were seeded in a 96-well plate and treated 48 h later with increasing concentrations of OTX015 alone or combined with crizotinib at a range of concentrations. GI_20_, GI_40_, GI_60_ and GI_80_ drug concentrations were evaluated. To assess drug-drug interactions, dose-response data were evaluated with the CalcuSyn program (Biosoft) which applies median effect methodology to estimate a combination index (CI), CI values < 0.9 indicate synergism, 0.9 to 1.1 indicate additive effects, and > 1.1 indicate antagonism [58].

## SUPPLEMENTARY MATERIALS FIGURES AND TABLES








