# Supplementary material for: OTX015 (MK-8628), a novel BET inhibitor, exhibits antitumor activity in non-small cell and small cell lung cancer models harboring different oncogenic mutations
Source: Oncotarget. 2016 Nov 7;7(51):84675–87. doi: 10.18632/oncotarget.13181 (PMC5354535; doi:10.18632/oncotarget.13181)
Supplement: Supplementary file 1 [file oncotarget-07-84675-s001.pdf]

OTX015 (MK-8628), a novel BET inhibitor, exhibits antitumor activity in non-small cell and small cell lung cancer models harboring different oncogenic mutations

Supplementary Materials

Gene mutation analysis

Genomic DNA was extracted using standard protocols. Briefly, cells were digested overnight with proteinase K, followed by standard ethanol precipitation. Genomic DNA was then eluted and resuspended in TE buffer. Exome capture was carried out using Illumina's TruSeq technology according to the manufacturer's protocols. Enriched exome libraries were then subjected to next generation sequencing using standard TruSeq sample preparation protocols from the manufacturer, and paired end sequencing was carried out on an Illumina HiSeq. Image analysis and base calling was carried out using CASAVA 1.8.2. BWA-MEM [1] was used to align sequence reads to the reference genome B37 (http://www.ncbi.nlm.nih.gov/projects/genome/assembly/grc/human/) with subsequent processing by Samtools (http://samtools.sourceforge.net) and Picard (http://broadinstitute.github.io/picard/) to ensure proper file formatting. Alignments were then recalibrated and realigned using GATK [2, 3]. To detect genome variants, GATK2 AtlasSNP version v1.4.3 [4], Samtools version 0.1.19 and FreeBayes version 0.9.7 were used. Integration of predicted small nucleotide polymorphisms (SNPs) from these algorithms was performed using BAYSIC [5]. Effect of SNPs and INDELs on genes was predicted using snpEff [6] with the GRCh37.69 database. Allele frequency in the general population was determined by comparison with the 1000G, the 100 Genome Consortium 2012 and EVS (http://evs.gs.washington.edu/EVS) databases. Additionally discovered variants are annotated using the SnpSift dbSNP (http://www.ncbi.nlm.nih.gov/snp/) and COSMIC [7] databases.

MTT assay

Cell proliferation was determined using the MTT assay. Cells were seeded in 96-well tissue culture plates at 20 × 10^3 cells/well. After OTX015 or JQ1 exposure (from 0.9 nM to 6 µM) for 72 h, cells were incubated with 0.4 mg/ml MTT for 4 h at 37°C. The cell pellet was then resuspended in 0.1 mL DMSO, and absorbance was measured at 560 nm using a microplate reader MultiSKan EX (Thermo Scientific). 0.1% DMSO was used as a negative control after establishing that it did not affect cell proliferation (data not shown). Drug potency was determined as the drug concentration at which cell proliferation is reduced by half (GI_{50}) and drug efficacy (E_{max}) as the percentage of cell proliferation at the highest OTX015 dose administered (6 µM), calculated with the equation for sigmoidal dose response using Prism 5.00 for MS Windows software (Graph Pad Software). Results are expressed as the mean and 95% confidence interval (95% CI) of at least three independent experiments performed in triplicate, unless otherwise indicated.

Real-time reverse transcriptase PCR

A total of 2 × 10^6 cells or ~10 mg of tumor samples were lysed in RTL buffer (Qiagen) and RNA was extracted using 70% ethanol and eluted using an RNeasy Mini spin column. Purity was determined using NanoDrop (NanoDrop Technologies). Reverse transcription was performed using a High Capacity cDNA Reverse Transcription Kit (Invitrogen, Fisher Scientific), and quantitative RT-PCR (qPCR) using FastStart Universal SYBR Green Master (Roche Diagnostics) according to the manufacturer's instructions. Primers were designed for BRD2, BRD3, BRD4, CDKN1A, BCL2, HIST2H2BE, HIST2H4A, HIST1H2BK, HIST1H2BJ, SESN3, HEXIM1, MTHFD1L, MYC, MYCN, CD44, CD24, CD133, EpCAM, Musashi-1, NANOG, OCT4, HPRT1, and GAPDH (Supplementary Table S1). In brief, FastStart Universal SYBR Green Master Mix (ROX) and nuclease-free water was added to cDNA (10 ng) in a 96-well PCR microplate. Cycles were 10 min at 95°C, then 40 cycles of 15 s at 95°C, then 60 s at 60°C, then a final cycle of 15 s at 95°C followed by 15 s at 60°C and 15 s at 95°C using a Mastercycler ep realplex 2 (Eppendorf). Relative quantification of gene expression was determined using the geometric mean method, normalizing target gene expression to two reference housekeeping genes (GAPDH and HRPT1).

Western blotting

Cells were solubilized in RIPA Lysis Buffer supplemented with protease and phosphatase inhibitors (Callbiochem, Merck KGaA). Lysates (20 µg) were fractionated by SDS-PAGE using Mini-Protean TGX

4–15% then transferred to a nitrocellulose membrane using a Trans-blot Turbo Starter system (BIO-RAD). Membranes were blocked for 1 h with 5% nonfat dry milk in TBS-T (0.05% Tween) then incubated with primary antibody in 5% nonfat dry milk in TBS-T overnight. Antibodies BRD2 (ab139690)and BRD3 (ab56342) were obtained from Abcam, N-MYC antibody (sc-53993) was obtained from Santa Cruz Biotechnology, and C-MYC (#5605), BCL2 (#2870), p21 Waf1/Cip1 (#2947), p-tyr705STAT3 (#9145), horseradish peroxidase (HRP)-conjugated anti-rabbit IgG (#7074) and anti-mouse IgG (#7076) were obtained from Cell Signaling. β-actin (#3700) was used as a loading control. Proteins were visualized using the SuperSignal West Pico Chemiluminescent Substrate ECLplus Western Blotting Detection Kit (Thermo Scientific), captured on a Chemi-Smart 2000 detection device and analyzed using Bio-Profil software (Vilber Lourmat).

Statistical analysis

Results are expressed as mean ± standard deviation (SD) of at least three independent experiments. Statistical analysis was performed by one-way ANOVA followed by Dunnett's Multiple Comparison Test, Student-Newman-Keuls test or by two-way ANOVA followed by Bonferroni, posteriori test employing Prism 5.00 for MS Windows. For the correlation studies, the strength of the linear association between two variables was quantified using the correlation coefficient r; r^2 represents the square of the correlation coefficient in linear regression.

REFERENCES

1. Li H. Aligning sequence reads, clone sequences and assembly contigs with BWA-MEM. arXiv 2013; 1303.3997.
2. DePristo M, Banks E, Poplin R, Garimella K, Maguire J, Hartl C, Daly M. A framework for variation discovery and genotyping using next-generation DNA sequencing data. Nature Genetics 2011; 43:491–498.
3. McKenna A, Hanna M, Banks E, Sivachenko A, Cibulskis K, Kernytsky A, DePristo M. The Genome Analysis Toolkit: a MapReduce framework for analyzing next-generation DNA sequencing data. Genome Research 2010; 20:1297–1303.
4. Challis D, Yu J, Evani US, Jackson AR, Paithankar S, Coarfa C, Milosavljevic A, Gibbs RA, Yu F. An integrative variant analysis suite for whole exome next-generation sequencing data. BMC Bioinformatics 2012; 13:8.
5. Cantarel BL, Weaver D, McNeill N, Zhang J, Mackey AJ, Reese J. BAYSIC: a Bayesian method for combining sets of genome variants with improved specificity and sensitivity. BMC Bioinformatics 2014; 15:104.
6. Cingolani P, Platts A, Wang le L, Coon M, Nguyen T, Wang L, Land SJ, Lu X, Ruden DM. A program for annotating and predicting the effects of single nucleotide polymorphisms, SnpEff: SNPs in the genome of Drosophila melanogaster strain w1118; iso-2; iso-3. Fly (Austin) 2012; 6:80–92.
7. Forbes SA, Tang G, Bindal N, Bamford S, Dawson E, Cole C, Kok CY, Jia M, Ewing R, Menzies A, Teague JW, Stratton MR, Futreal PA. COSMIC (the Catalogue of Somatic Mutations in Cancer): a resource to investigate acquired mutations in human cancer. Nucleic Acids Res. 2010; 38:D652–657.

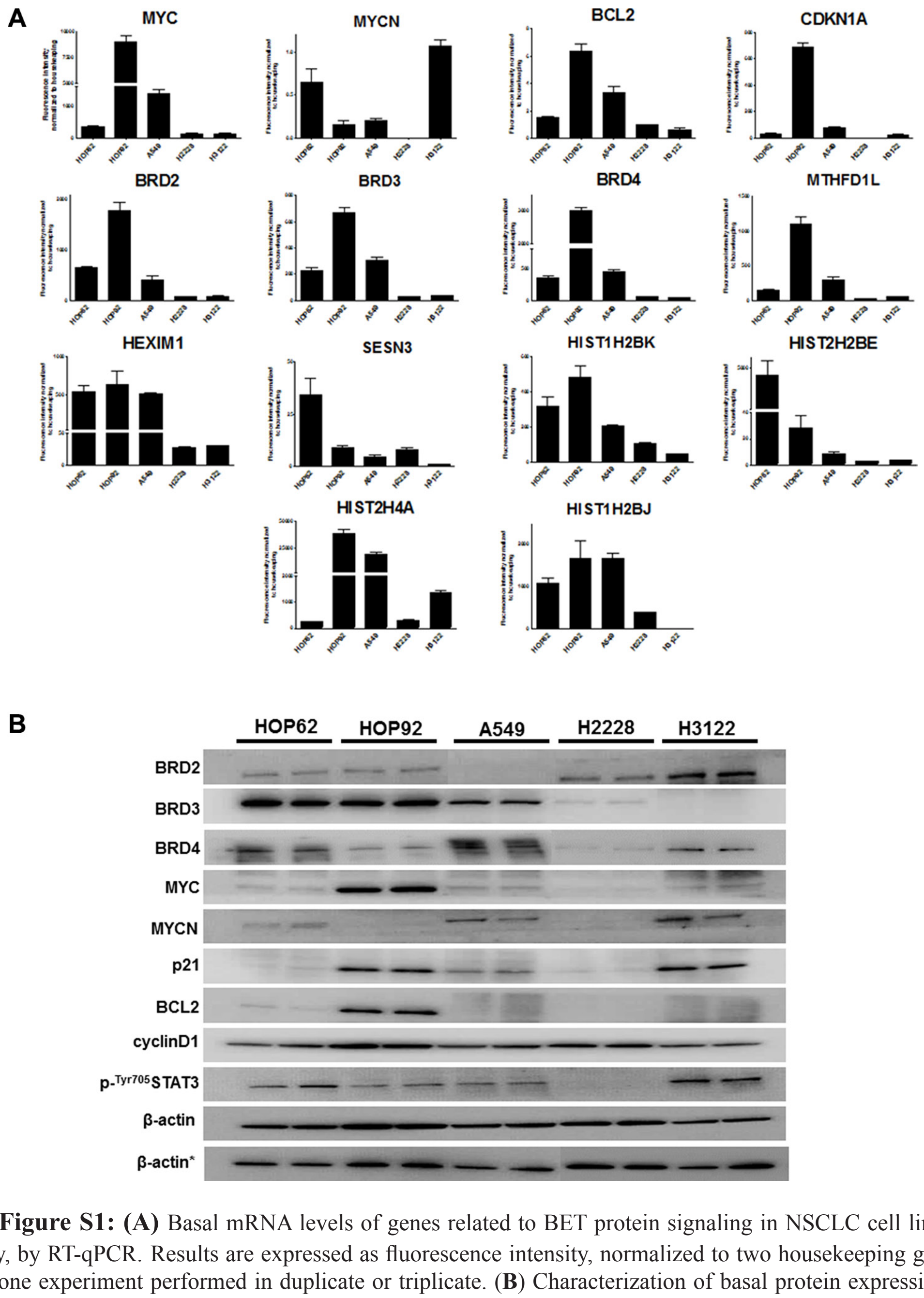

Supplementary Figure S1: (A) Basal mRNA levels of genes related to BET protein signaling in NSCLC cell lines with a range of OTX015 sensitivity, by RT-qPCR. Results are expressed as fluorescence intensity, normalized to two housekeeping genes, and represent the mean ± SD of one experiment performed in duplicate or triplicate. (**B**) Characterization of basal protein expression of MYC, BRDs and other cell survival and cell cycle-related pathway in NSCLC cell lines, by Western blot. Results are representative of at least two independent experiments, with duplicate lanes for each cell line. β-actin was used as a loading control. *This β-actin was used as a loading control only for BRD4 western blot.

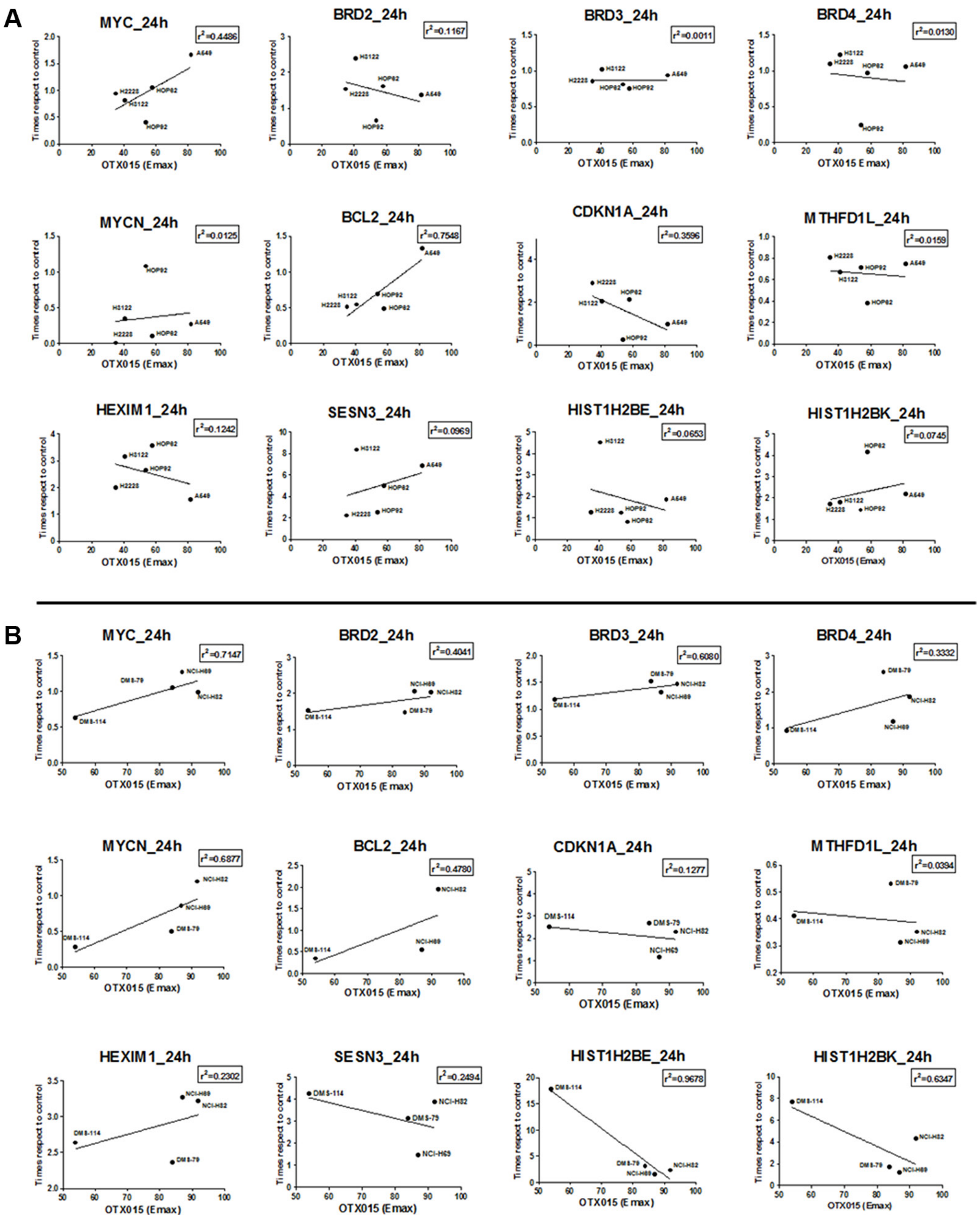

Supplementary Figure S2: OTX015 treatment modulates gene expression of several genes, but does not correlate with biological efficacy, in (A) NSCLC and (B) SCLC. Correlation between changes in MYC, MYCN, BCL2, CDKN1A, BRDs, MTHFD1L, HEXIM1, SESN3, HIST1H2BK, HIST2H2BE, HIST2H4A and HIST1H2BJ mRNA levels after 500 nM OTX015 for 24 h with OTX015 E_{max}. Fold changes are relative to 0.1% DMSO control. Correlation coefficients (r^2) are shown.

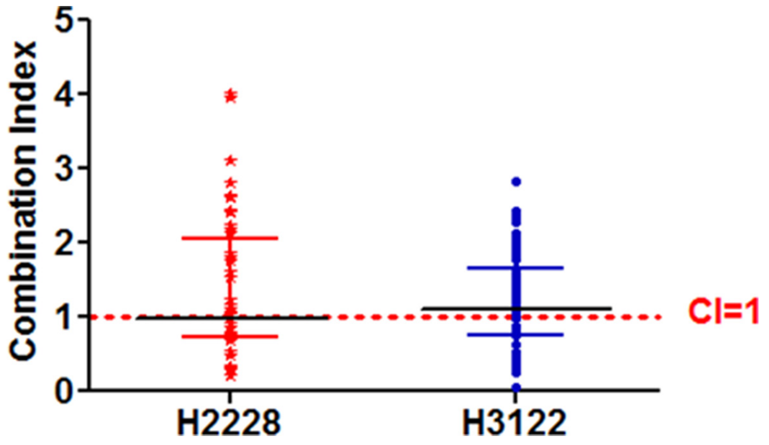

Supplementary Figure S3: OTX015 combination study with crizotinib in NSCLC-EML4-ALK(+) cell lines after 48 h concomitant exposure, by the Chou-Talalay method. Median CI values are CI = 0.9 (range, 0.2–4.0) and CI = 1.1 (range, 0.1–2.8) for H2228 and H3122, respectively. Results represent the median with interquartile range of four independent experiments performed in triplicate. CI values between 0.9 and 1.1 reflect an additive effect.

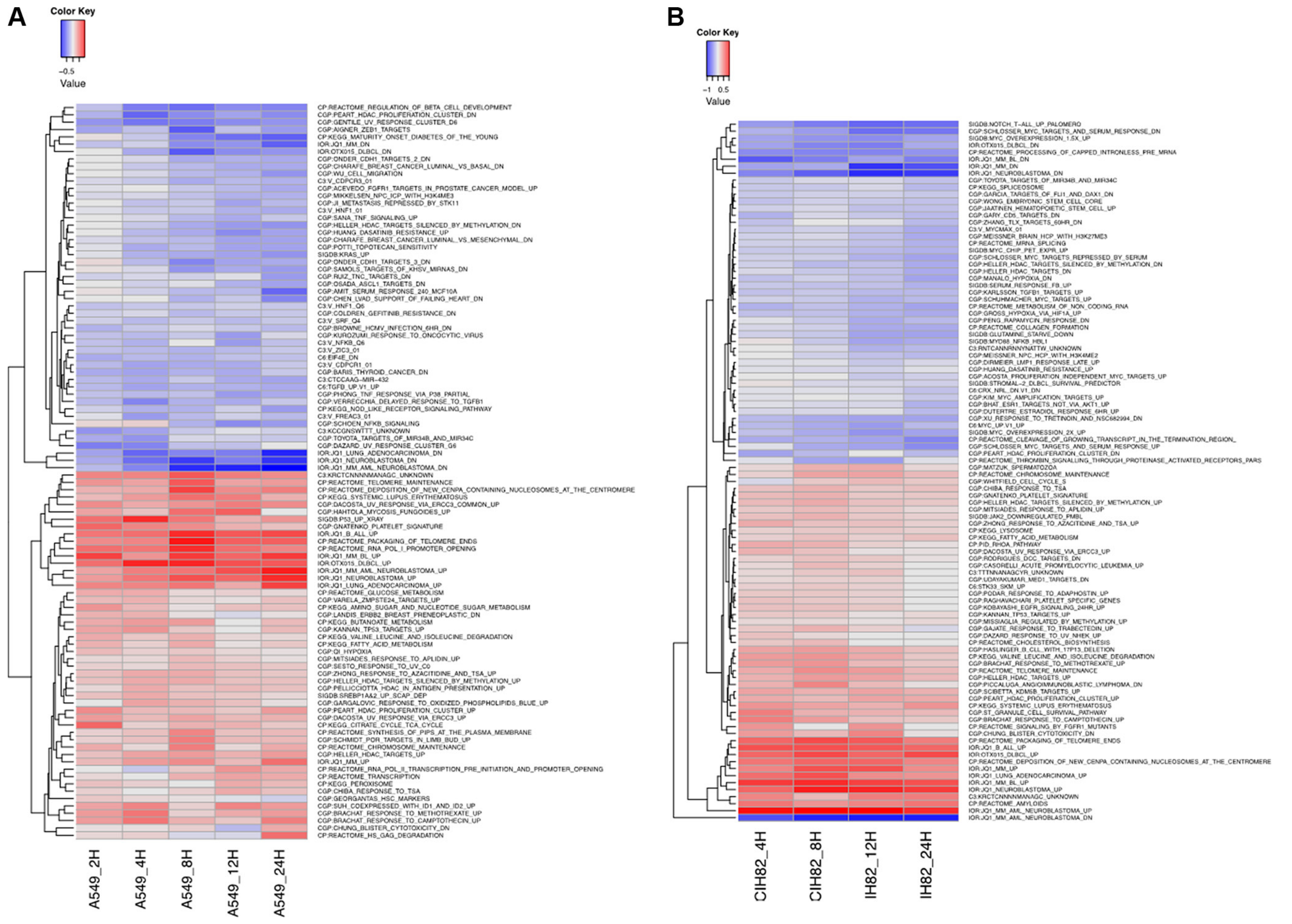

Supplementary Figure S4: Heatmap of average gene set expression of the most enriched gene sets among genes changing after exposure to OTX015 in lung cancer OTX015 resistant cell lines. (**A**) NSCLC cell lines; (**B**) SCLC cell line. The top 50 most enriched gene sets among upregulated genes and the top 50 enriched gene sets among the downregulated genes are shown. Gene set expression was calculated using GSVA. Y-axis: cell lines at different time points. The heatmap colors are not scaled and show the consistent increase (or decrease) of gene set expression in time. X-axis: top differentially expressed gene sets as assessed by GSEA. Gene sets were filtered for a minimal fold-change of > 0.15 and FDR < 0.05.

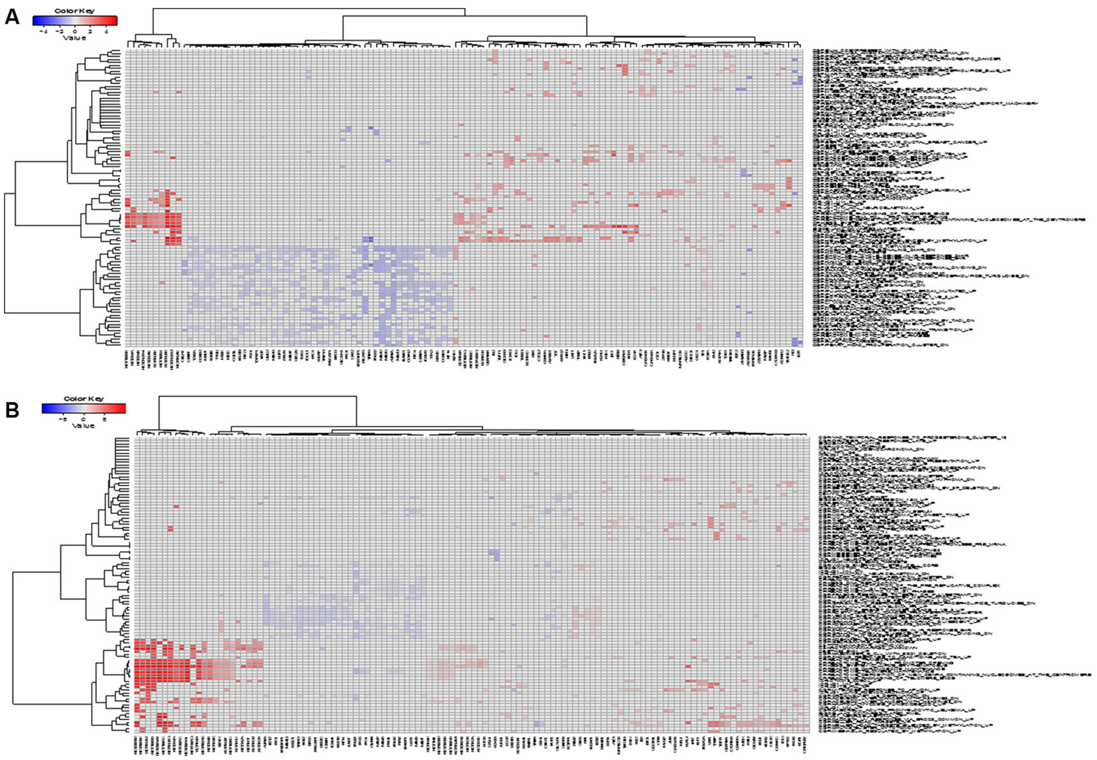

Supplementary Figure S5: Hierarchical clustering of the top genes in the top gene sets enriched in genes affected by OTX015 in OTX015-sensitive cell lines. (**A**) NSCLC cell lines; (**B**) SCLC cell line. The heatmap coloring shows the overall importance of a gene and gene set combination in the data set. The scale is proportional to the product of gene fold-change and the absolute normalized enrichment score (NES) from GSEA.

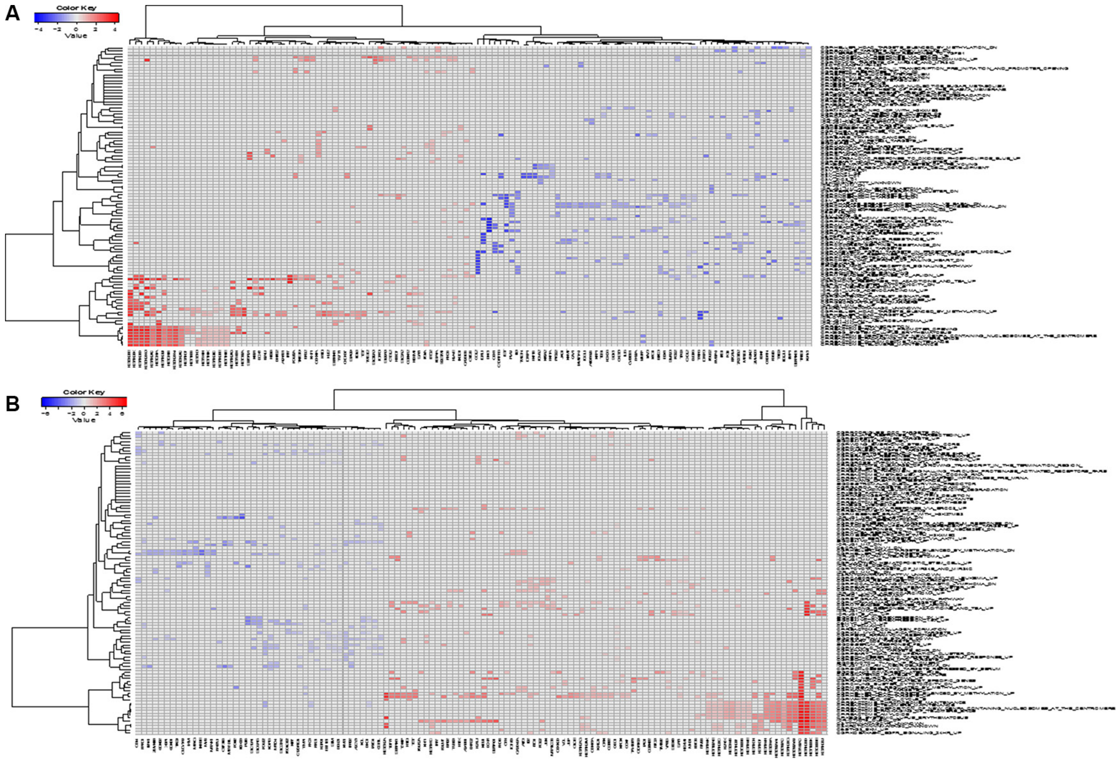

Supplementary Figure S6: Hierarchical clustering of the top genes in the top gene sets enriched in genes affected by OTX015 in OTX015-resistant cell lines. (**A**) NSCLC cell lines; (**B**) SCLC cell line. The heatmap coloring shows the overall importance of a gene and gene set combination in the data set. The scale is proportional to the product of gene fold-change and the absolute normalized enrichment score (NES) from GSEA.

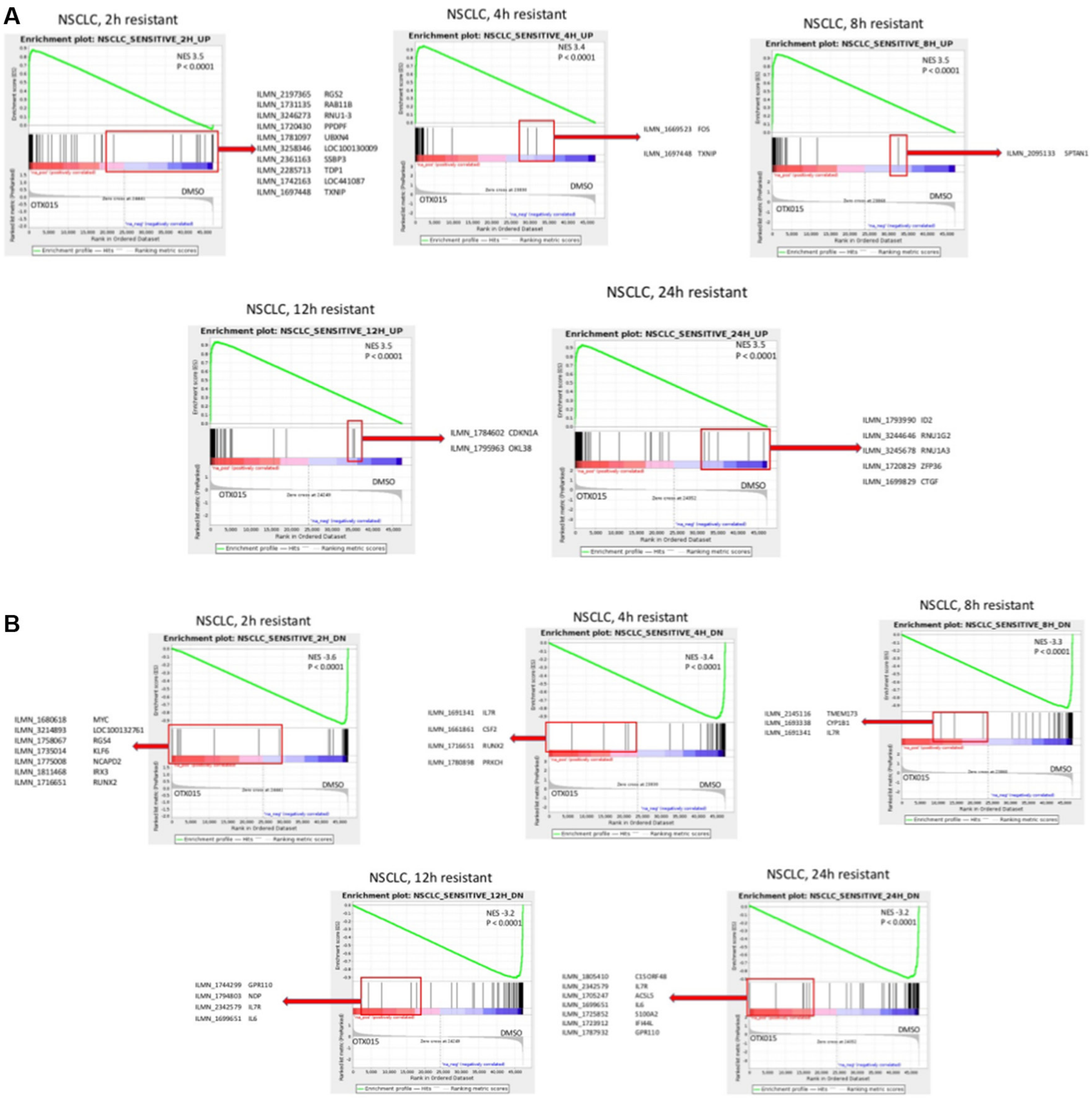

Supplementary Figure S7: NSCLC resistant cells treated with OTX015: GSEA plots of OTX015 signatures derived from OTX015-treated sensitive NSCLC cells. (**A**) Signatures of transcripts up-regulated by OTX015; (**B**) Signatures of transcripts down-regulated by OTX015. NES, normalized enrichment score. Boxes highlight probes that behaved differently between resistant and sensitive cells.

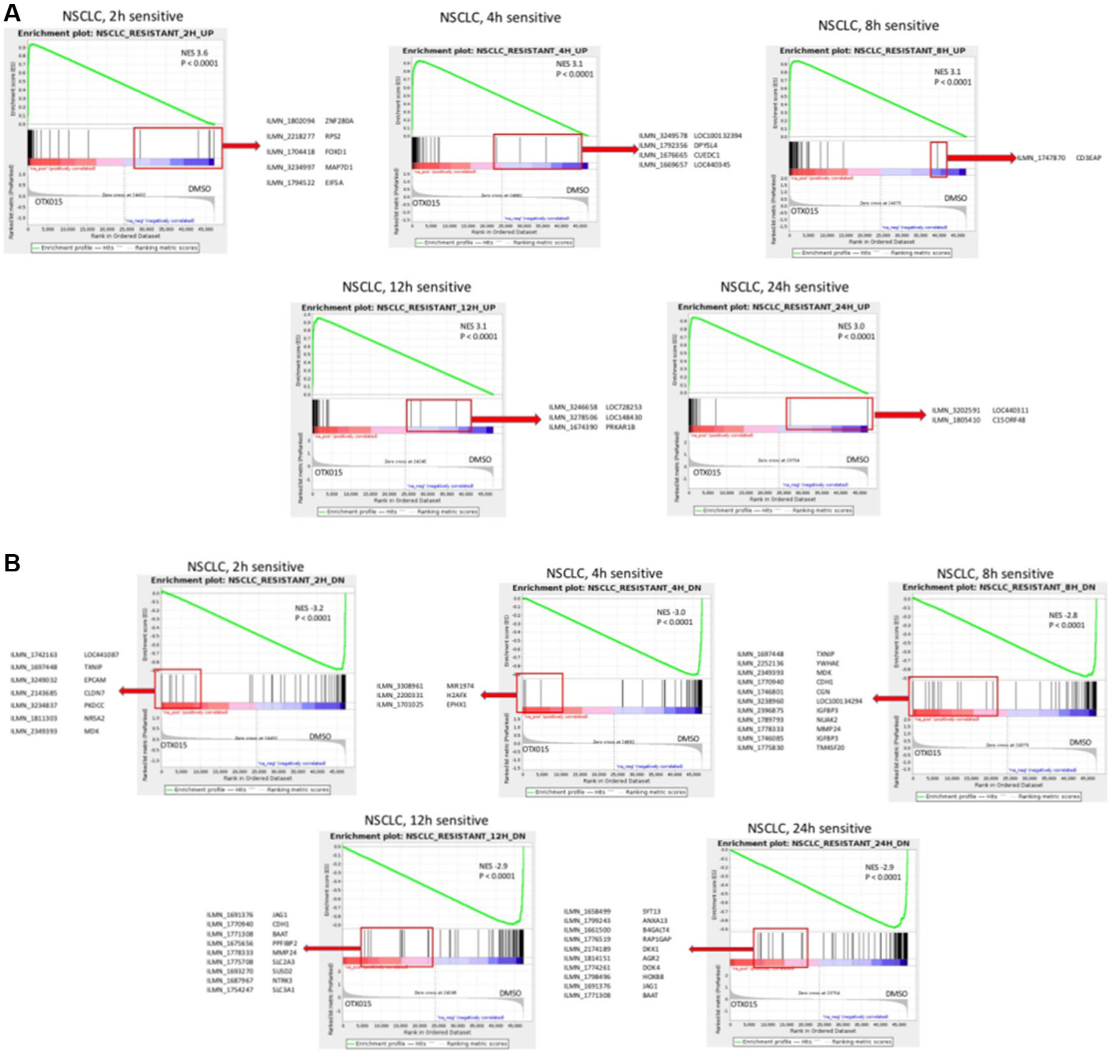

Supplementary Figure S8: NSCLC sensitive cells treated with OTX015: GSEA plots of OTX015 signatures derived from OTX015-treated resistant NSCLC cells. (**A**) Signatures of transcripts up-regulated by OTX015; (**B**) Signatures of transcripts down-regulated by OTX015. NES, normalized enrichment score. Boxes highlight probes that behaved differently between resistant and sensitive cells.

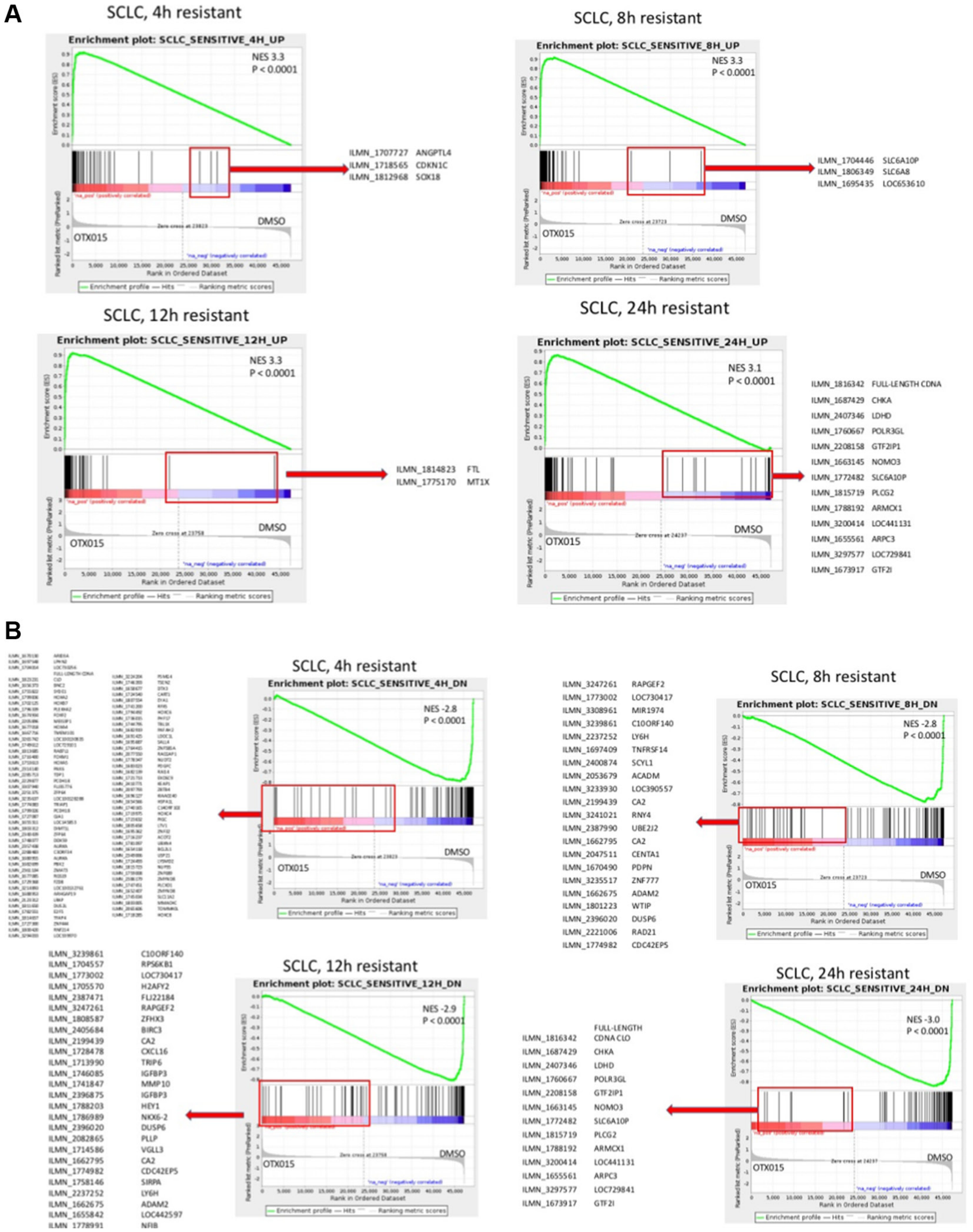

Supplementary Figure S9: SCLC resistant cells treated with OTX015: GSEA plots of OTX015 signatures derived from OTX015-treated sensitive SCLC cells. (**A**) Signatures of transcripts up-regulated by OTX015; (**B**) Signatures of transcripts down-regulated by OTX015. NES, normalized enrichment score. Boxes highlight probes that behaved differently between resistant and sensitive cells.

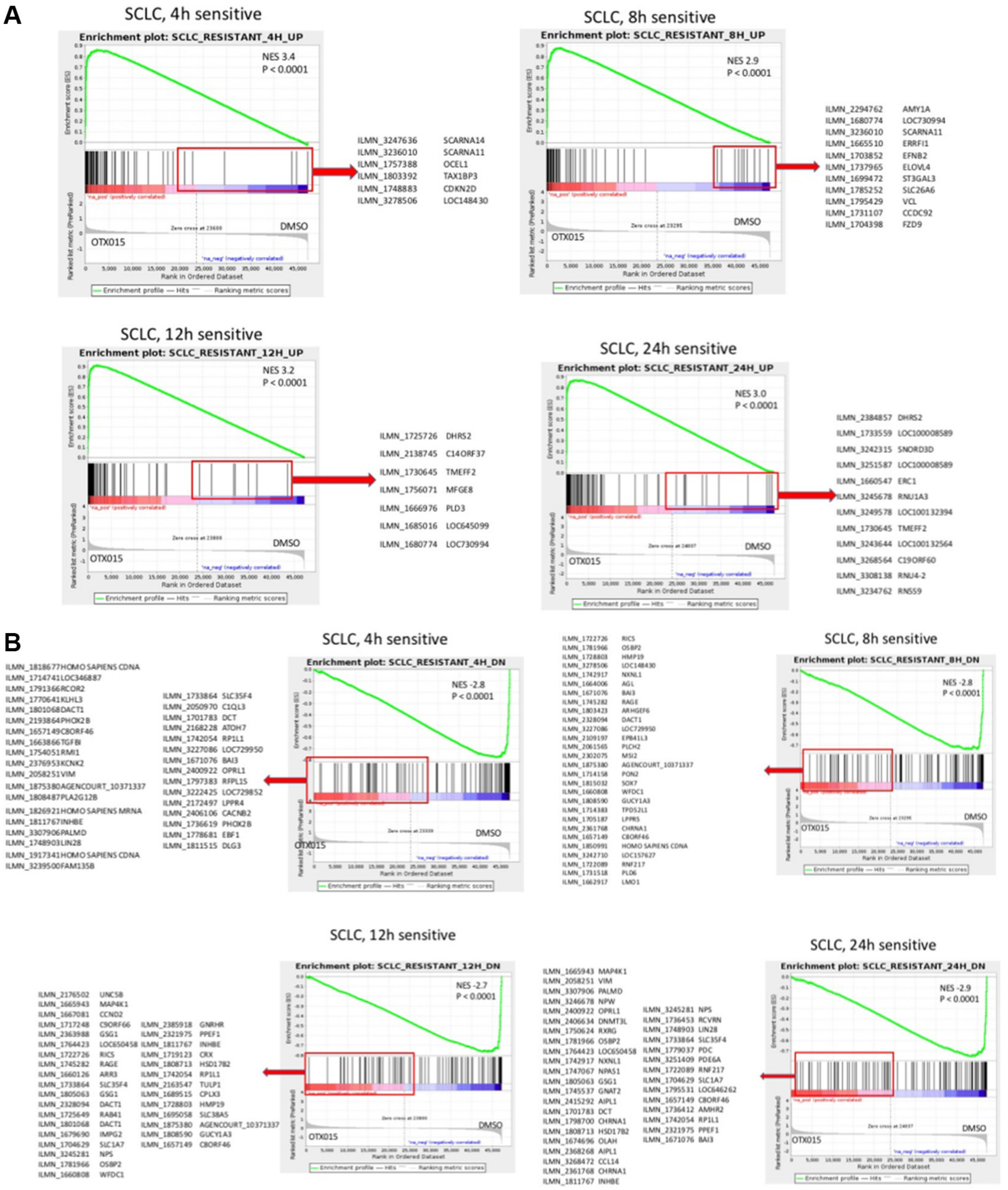

Supplementary Figure S10: SCLC sensitive cells treated with OTX015: GSEA plots of OTX015 signatures derived from OTX015-treated resistant SCLC cells. (**A**) Signatures of transcripts up-regulated by OTX015; (**B**) Signatures of transcripts down-regulated by OTX015. NES, normalized enrichment score. Boxes highlight probes that behaved differently between resistant and sensitive cells.

Supplementary Table S1: Primer sequences (forward and reverse) for qPCR studies

Genes	Primers	Sequences
BRD2	Fw	ccacctcaacctaagaagtcca
	Rv	acttcctccagaaggtccaaag
BRD3	Fw	gtgcacatcatccaatctcg
	Rv	cttgctgagaacggtttcct
BRD4	Fw	tctacaacaagcctggagatga
	Rv	tctcggtttcttctgtgggtag
MYC	Fw	cgactctgaggaggaacaagaa
	Rv	ggatagtccttccgagtgga
MYCN	Fw	tgagcgattcagatgatgaaga
	Rv	gcatcgtttgaggatcagc
CDKN1A	Fw	gaccagcatgacagatttctacc
	Rv	aagatgtagagcgggccttt
BCL2	Fw	tgatgggatcgttgcctta
	Rv	tccaattcctttcggatcttt
SESN3	Fw	tatgtttggaatcaggtatgatgac
	Rv	ggtcaaatgccgagttatgg
HEXIM1	Fw	aaggactagctaaaggcgtcac
	Rv	tggctagtagagtcctcgaagttt
MTHFD1L	Fw	aattatccaggcaggtgacg
	Rv	aatctcggcttcactgctatct
HIST2H4A	Fw	ctggcctcatttacgaggag
	Rv	gggatcgaaacgtgcaa
HIST2H2BE	Fw	cttgactccaaaggctcttttc
	Rv	tatgccatttcccatgacct
HIST1H2BJ	Fw	agctacacagtgctatgccaga
	Rv	gtctttcttctgcgccttagtc
HIST1H2BK	Fw	acttgccaaggagggacttt
	Rv	aaggcaattgtgcttcttttg
CD44	Fw	ccctccgtcttaggtcactg
	Rv	cggcaggttatattcaaatcg
CD24	Fw	tcctcccagagtacttccaact
	Rv	gagtgagaccacgaagagactg
CD133	Fw	cagctacttggctcagactgg
	Rv	gtgcatctcttttcagggagtt
EpCAM	Fw	ttgtggttgtggtgatagcag
	Rv	cacccatctcctttatctcagc
Musashi-1	Fw	tgttcatcgggggactca
	Rv	ggtcaattgttttggagtcgag
NANOG	Fw	gccttgctttgaagcatcc
	Rv	gaggaaggaagaggagagacagt
OCT4	Fw	cacacaaagcactttatccattct
	Rv	tcacccccagtttaaggatgt
GAPDH	Fw	gatccctccaaaatcaagtgg
	Rv	ggaggcattgctgatgatct
HPRT1	Fw	tgaatacttcagggatttgaatcat
	Rv	ctcatcttaggctttgtattttgc

Supplementary Table S2: Complete characterization of *KRAS*, *EGFR, PIK3CA*, *LKB1*, *TP53* and *RB1* status in our panel of NSCLC and SCLC cell lines

Cell lines		KRAS Exon2	PIK3CA Exon20	LKB1	EGFR	TP53	RB1
NSCLC	HOP62	Heterozygous mutation, Missense variant, c.34G > A, p.Gly12Ser	WT	WT	WT	4 homozygous mutations, Splice acceptor variant&Intron variant, c.673-2A >G ; n.555-2A > G; c.277-2A >G ; c.394-2A > G ; n.68-2A>G	WT
	HOP92	WT	WT	WT	WT	3 homozygous mutations, Missense variant, c.524G > T p.Arg175Leu ; c.128G > T p.Arg43Leu ; c.245G >T p.Arg82Leu	WT
	A549	Homozygous mutation, Missense variant, c.34G > T, p.Gly12Cys	WT	Homozygous mutation, Stop gained, c.109C > T, p.Gln37*	WT	WT	WT
	NCI-H2228	WT	WT	WT	WT	3 homozygous mutations, Stop gained, c.991C > T p.Gln331* ; c.49C > T p.Gln17* ; c.595C > T p.Gln199*	Homozygous mutation, Deletion in frame, c.610delG, p.Glu204fs
	NCI-H3122	WT	WT	WT	WT	2 homozygous mutations, Missense variant, c.854A > T p.Glu285Val ; c.458A > T p.Glu153Val	WT
SCLC	NCI-H69	WT	Heterozygous mutation, Deletion in frame, c.317_325del9, p.Gly106_Arg108del	WT	WT	3 homozygous mutations, Stop gained, c.511G > T p.Glu171* ; c.115G > T p.Glu39* ; c.232G > T p.Glu78*	Homozygous mutation, Stop gained, c.2242G > T, p.Glu748*
	NCI-H82	WT	WT	WT	WT	WT	Homozygous mutation, Splice acceptor variant&Intron variant, c.940,2A > T
	DMS79	WT	WT	WT	WT	4 deletion in frame: 2 heterozygous mutation, c.838delA p.Arg280fs and c.442delA p.Arg148fs; and 2 homozygous mutations, c.834_835delTGinsA p.Gly279fs and c.438_439delTGinsA p.Gly147fs	Homozygous mutation, Stop gained, c.2401G > T, p.Gly801*
	DMS114	WT	WT	WT	Heterozygous mutation, Stop gained, c.1379C > A, p.Ser1060*	3 homozygous mutations, Stop gained, c.637C > T p.Arg213* ; c.241C > T p.Arg81* ; c.358C > T p.Arg120*	WT

Moderate impact (pink) **High impact** (grey)

Supplementary Table S3: Supervised analysis of the gene expression profiles of NSCLC cell lines after OTX015 exposure as obtained by limma. See Supplementary_Table_S3.

Supplementary Table S4: OTX015 gene expression signatures obtained in lung cancer cell lines exposed to OTX015 or DMSO at different time points. The signatures comprise the top 100 most differentially expressed transcripts between OTX015 and control cells. See Supplementary_Table_S4.

Supplementary Table S5: Supervised analysis of the gene expression profiles of SCLC cell lines after OTX015 exposure as obtained by limma. See Supplementary_Table_S5.
